# Internal and external load during 8 v 8, 5 v 5 and 3 v 3 in Chinese elite youth male football players

**DOI:** 10.5114/biolsport.2022.113292

**Published:** 2022-02-04

**Authors:** Zhen Li, Lijuan Mao, Peter Krustrup, Morten B. Randers

**Affiliations:** 1 Department of Physical Education and Sports Training, Shanghai University of Sport, Shanghai, China; 2 Department of Sports Science and Clinical Biomechanics, SDU Sport and Health Sciences Cluster (SHSC), University of Southern Denmark, Odense, Denmark; 3 School of Sport Sciences, Faculty of Health Sciences, UiT The Arctic University of Norway, Tromsø, Norway

**Keywords:** Small-sided games, Game formats, GPS, Sprints, High-intensity running, Blood lactate

## Abstract

Aim to investigate internal and external load in three different game formats (8 v 8, 5 v 5, 3 v 3 with 80 m^2^ per player) of small-sided games (SSG) in Chinese elite youth football players. Twenty-nine elite male football players (age: 18.3 ± 0.5 years (mean ± SD), height: 175 ± 6 cm, weight: 65.5 ± 6.3 kg) participated in randomized order in the three formats. Each session consisted of 20 min: 3 v 3 on a 24 × 20-m pitch, 5 v 5 on a 32 × 25-m pitch, or 8 v 8 on a 40 × 32-m pitch all equalling 80 m^2^ per player. Each player was recorded once in each format. Using GPS-units and heart rate belts and blood lactate measured the two kinds of load. 8–10% higher total distance (P < 0.01) was observed in 8 v 8 and 5 v 5 compared with 3 v 3 (1627 ± 240 and 1595 ± 243 m vs. 1477 ± 179 m, ES = 0.55–0.71). Higher distance (P < 0.001) was covered with high speed running (HSR: > 14 km/h) in 8 v 8 and 5 v 5 than 3 v 3 (154 ± 94 m and 133 ± 59 m vs. 77 ± 35, ES = 1.09–1.15), whereas very high speed running distance (> 21 km/h) was higher (P < 0.01) in 8 v 8 than 5 v 5 and 3 v 3 (15.2 ± 19.5 vs. 5.3 ± 6.7 and 1.0 ± 0.4 m, ES = 0.69–1.03) and in 5 v 5 than 3 v 3. No difference was found between game formats in the number of intense accelerations nor intense decelerations. Blood lactate (3.5 ± 2.3 vs. 2.8 ± 1.9 vs. 2.4 ± 1.5 mmol · L^-1^, P = 0.201) and mean heart rate (155 ± 21 vs. 160 ± 11 vs. 157 ± 17 bpm, P = 0.254) was not different between 8v8, 5v5 and 3v3 game formats. Distance covered in total and in highest speed zones was higher in SSG formats with more players, which, however, did not lead to differences in internal load measured by heart rate and blood lactate.

## INTRODUCTION

Small-sided games (SSG) have been shown to be effective means to improve physical fitness [1, 2] and are favoured over interval running for maintaining and improving fitness along with technical abilities as SSG are considered more enjoyable [3] even though studies have shown that running-based drills may be more effective in improving fitness [4, 5]. Moreover, SSG have traditionally been considered to be a more relevant method for improving fitness for football players due to the movement specificity [6], but recent studies have highlighted points to pay attention to as the coefficient of variation (CV) for high-speed running in SSG are large [7] and the relation to match demands may be questioned [8]. Nevertheless, SSG are widely used during training for preparation of physical as well as technical-tactical abilities [9].

To maximize the physiological response to the applied training, coaches must be aware of the load applied to the players through the planned exercises. Even though monitoring load often focuses on external load, due to practicality, and estimates of internal load are based on these measures of external load, a direct relationshipbetween external load and internal response cannot be expected [10]. In SSG, external and internal load can be manipulated by changing task constraints such as number of players, pitch size, goal size, game rules, duration, etc. [6, 9, 11]. Hence, it is essential to understand how these manipulations affect external and internal load.

Several studies have been conducted to investigate the effect of changes in game format [6, 11–19]. For example, it has been shown that more high-speed running is performed, and higher peak speed is reached on larger pitches, which has been found both in studies with a constant area per player [13, 15] and those with differences in area per player [17]. Other studies have, however, shown that the relative pitch area is an important factor, as no difference in peak speed or distance covered at high speed was observed when area per player was kept constant [15]. Peak speed and distance covered at high speed increased, however, with increasing area per player when pitch size was kept constant and area per player was increased by decreasing the number of player [16]. Thus, the literature lacks a consensus on the effects of changing the number of players and size of the pitch on external load of various game formats. Similar, contradictory responses of changing the pitch size and number of players have been found for internal load, most often measured as heart rate and blood lactate [13, 15, 16, 18, 19]. In addition, a considerable number of these studies have been conducted on amateur or recreational players [15, 16, 19]; thus well-designed studies on elite players are scarce.

Moreover, the relation between external load in various SSG formats and matches has been questioned [8], and whether external and internal load variables are correlated between various SSG formats is not well investigated.

Therefore, the aim of this study was to investigate the effect of three different game formats of SSG (8 v 8, 5 v 5, 3 v 3) on internal and external load in Chinese elite youth football players and whether these external and internal load variables are correlated between the three game formats.

## MATERIALS AND METHODS

### Experimental approach

Twenty-nine Chinese male U-19 football players from a professional football academy participated in randomized order in three formats of SSG. All players without any injuries from the squad were included and characteristics are presented in Table 1. Data were collected for each player once per game format. Three sessions were carried out with one day apart in the same week (3^rd^ to 7^th^ of August 2019) during the initial part of the preparation period; thus no fatigue from matches or other competitive sessions was experienced. Weather conditions were similar on all three session days (~30°C, 70% humidity, partly cloudy and light to moderate breeze).

**TABLE 1:** Descriptive data of the participants (mean ± SD).

	Mean ± SD
Age (years)	18.3 ± 0.5
Height (cm)	175 ± 6
Weight (kg)	65.5 ± 6.3
BMI (kg/m^2^)	21.5 ± 1.7
Fat percentage (%)	8.7 ± 3.2
10-m sprint (s)	1.528 ± 0.095
30-m sprint (s)	4.021 ± 0.166
Standing long jump (m)	1.90 ± 0.12
Yo-Yo IR1 distance (m)	1434 ± 315

Prior to the SSG sessions, the participants were familiarized with the SSG formats on one to three occasions. Players were tagged according to their usual playing position in matches as goal keepers (n = 3), defenders (n = 10), midfielders (n = 14) and forwards (n = 2). For position-specific analysis, midfielders and forwards were merged to M/F and defenders as the separate group D, whereas goalkeepers were excluded.

Before being enrolled in the study, all participants were fully informed of all procedures and possible risks before giving written informed consent to participate. The study was conducted in accordance with the Declaration of Helsinki and approved by the local ethics committee (Shanghai University of Sport).

### Testing procedure

Before completing the different SSG, all participants completed a test battery organised by the main author encompassing standing and sitting height, weight, body composition, standing long jump, 10-m and 30-m sprint test and Yo-Yo IR1 test performance. The anthropometry tests were conducted about one hour before the starting training session in the morning and all participants were divided into four groups to conduct the tests in rotation. The physical fitness tests were carried out two days before the first SSG session.

Height was measured using a Tanita Leicester Height Measure, followed by weight and fat percentage measurement (Inbody 770, South Korea).

Standing long jump performance was evaluated in the inside gym giving each player two trials of standing forward jump with hands on the waist. Before the start of the test, the participants were allowed to try one time for familiarization. The test result was recorded as the distance measured from the start line to the point nearest to the first landing position. The two trials were separated by a 2-min rest and the best of the two trials was recorded as the test result.

Sprint performance was evaluated on a synthetic field from a standing start over distances of 10 and 30 m using electronic timing gates (SMARTSPEED PRO, Fusion Sport, Australia), which use the principle of infrared light to perform a segmented timing test for athletes’ speed with an accuracy of 1 millisecond. Two trials were applied separated by a 3-min rest interval and the best trial at each distance was recorded as the test result.

Following the other tests, a Yo-Yo Intermittent Recovery test level 1 (Yo-Yo IR1) was conducted after 15 min of rest and a 15-min long warm-up. The Yo-Yo IR1 test consists of repeated 2 × 20-m running at increasing speeds dictated by audio bleeps interspersed by 10-s active resting intervals as described by Krustrup and colleagues [20]. All subjects had been familiarized with the Yo-Yo IR1 test prior to the testing period by carrying out the full test including warm-up.

### Small-sided games (SSG)

After a standardized warm-up the 20 min SSG sessions were completed. The three SSG formats were 3 v 3 on a 24 × 20-m pitch, 5 v 5 on a 32 × 25-m pitch, and 8 v 8 on a 40 × 32-m pitch; thus in all game formats area per player was 80 m^2^ with a length:width ratio of 1.20–1.28. The size of the goals was 3 × 2-m for 3 v 3/5 v 5 and 5.5 × 2-m for 8 v 8. The participants completed three different SSG and the SSG were played with one-third of the participants starting with 3 v 3 and ending with 8 v 8, another one-third starting with 8 v 8 and ending with 3 v 3, and the last one-third participants starting with 5 v 5, then 8 v 8 and ending with 3 v 3. In a randomized order, 1/3 of the participants completed on each session day all three SSG formats interspersed with 5 minutes of passive rest.

During all SSG sessions plenty of balls were available to restart the game when the ball was out of play. Moreover, players were verbally encouraged by the coach to maintain a high pace during all sessions.

### Movement pattern

All players wore GPS units (Minimax S4, Catapult Innovations, Australia) sampling at a rate of 10-Hz, which have been shown to be valid and reliable to measure football player movements [21, 22]. A GPS unit was placed into a harness on the player’s upper back as described by the manufacturer. To limit inter-unit variability, participants wore the same unit during all three sessions. A similar number of satellites and similar mean horizontal dilution of precision were observed in the three sessions. Maximal speed, total distance covered, and distances covered at 0–7, 7–14, 14–21, 21–24, and > 24 km · h^-1^ were measured. Speed zones > 14 km · h^-1^ were summarised as high-speed running (HSR), whereas speed zones > 21 km · h^-1^ were summarised as very high-speed running (VHSR). Moreover, maximal acceleration and deceleration as well as number of intense accelerations (> 2 m s^-2^) and intense decelerations (< -2 m s^-2^) were measured.

### Heart rate monitoring

Heart rate was monitored at 1-s intervals using short range radio telemetry (Polar Team Pro, Polar Electro Oy, Kempele, Finland). Mean and peak heart rate are presented as absolute values and relative to the highest individual heart rate observed during the three sessions of SSG or the Yo-Yo IR1 test.

### Blood lactate

Blood lactate was measured within 3 min after the 20 min SSG using sensor sticks (LactateScout+, EKF, German). A drop of blood was transferred from the index finger after sterilization using an alcohol wiper and piercing the skin using a scalpel. The lactate sticks were analysed using LactateScout Sensors (Code 36, EKF, German).

### Statistics

Data are presented as means ± SD. Data were checked for normality using the Shapiro-Wilk test and for equal variance using Brown-Forsythe test. A few variables were not normally distributed and were therefore log-transformed. Differences in movement pattern, heart rate and blood lactate were evaluated using one-way analysis of variance with repeated measures (ANOVA RM). When a significant interaction was detected, data were subsequently analysed using the Student-Newman-Keuls post hoc test. A significance level of 0.05 was chosen. Effect sizes (ES) were calculated as differences in means divided by the pooled SD [23] and interpreted as suggested by Hopkins and colleagues [24]: < 0.2 trivial, 0.2–0.6 small, 0.6–1.2 moderate, 1.2–2.0 large. Correlations were tested using Pearson’s correlation coefficient and interpreted as described by Hopkins and colleagues [24].

## RESULTS

### Movement pattern

**FIG. 1:**
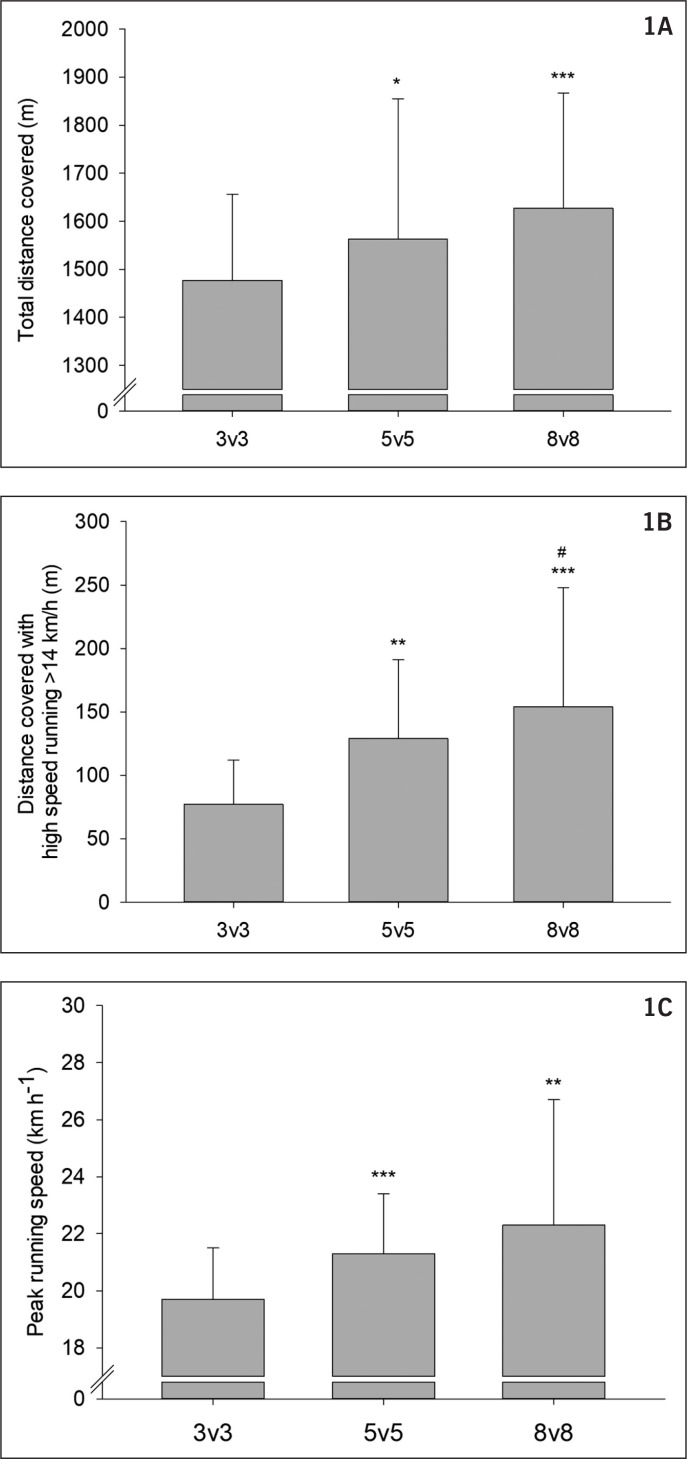
A) Total distance covered, B) distance covered with high-speed running (HSR) with speed > 14 km · h^-1^ and C) peak running speed during 3 v 3, 5 v 5 and 8 v 8. * denotes significantly different from 3 v 3 and # denotes significantly different from 5 v 5. Number of symbols denotes the degree of significance, e.g. *, **, *** P < 0.05, P < 0.01, P < 0.001, respectively.

The total distance covered was 8–10% longer during 8 v 8 and 5 v 5 compared to 3 v 3 (1627 ± 240 and 1595 ± 243 m vs. 1477 ± 179 m, P < 0.01, ES = 0.71 and ES = 0.55, respectively) with no difference between 8 v 8 and 5 v 5 (P = 0.196 , Fig. 1A). A greater distance was covered with HSR in 8 v 8 and 5 v 5 than in 3 v 3 (154 ± 94 and 133 ± 59 vs. 77 ± 35 m, P < 0.001, ES = 1.09 and ES = 1.15, respectively) with no significant difference between 8 v 8 and 5 v 5 (P = 0.092 , Fig. 1B). Moreover, a greater distance was covered with VHSR in 8 v 8 than 5 v 5 and 3 v 3 (15.2 ± 19.5 vs. 5.3 ± 6.7 and 1.0 ± 2.2 m, P < 0.05 and P < 0.001, ES = 0.69 and ES = 1.02, respectively) and in 5 v 5 than 3 v 3 (P = 0.003, ES = 0.85).

Higher peak speed (P < 0.001) was found in 8 v 8 and 5 v 5 than in 3 v 3 (22.3 ± 4.4 and 21.4 ± 2.0 vs. 19.7 ± 1.8 km · h^-1^; P < 0.001 and P < 0.01, ES = 0.77 and ES = 0.89, respectively), with no difference between 8 v 8 and 5 v 5 (P = 0.130 , Fig. 1C).

No difference was found between 8 v 8, 5 v 5 and 3 v 3 in the number of intense accelerations (12.9 ± 6.8 vs. 14.7 ± 6.0 vs. 12.2 ± 6.2; P = 0.148, respectively) or intense decelerations (13.8 ± 7.4 vs. 15.9 ± 6.6 vs. 14.2 ± 6.9, P = 0.282, respectively).

### Heart rate and blood lactate

**FIG. 2:**
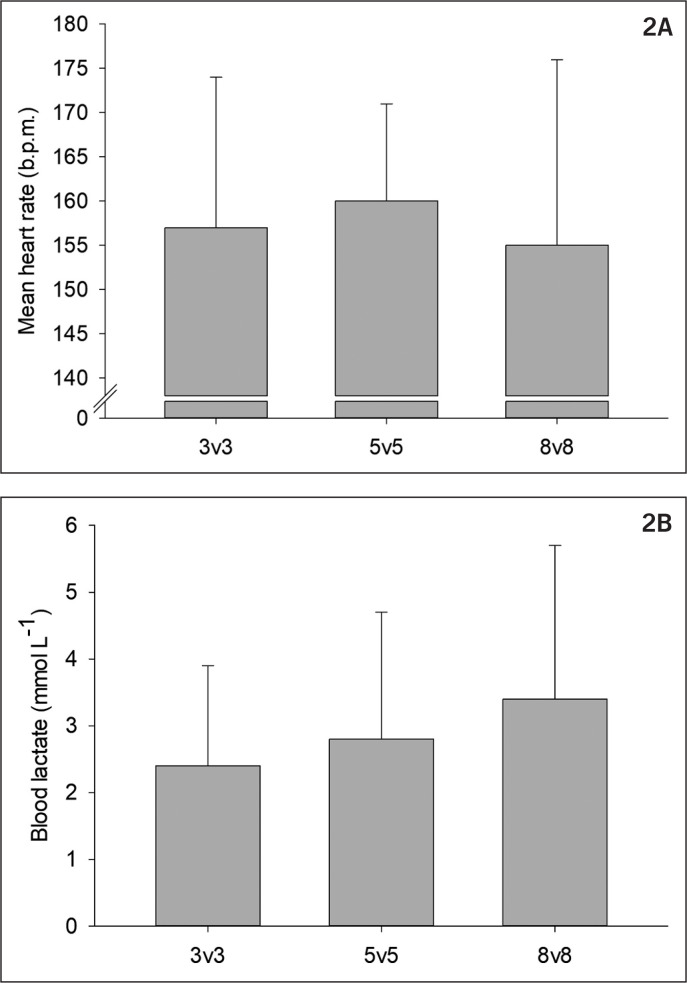
A) Mean heart rate and B) blood lactate concentration during 3 v 3, 5 v 5 and 8 v 8.

No difference was found in mean heart rate (155 ± 21 vs. 160 ± 11 vs. 157 ± 17 bpm, P = 0.254 , Fig. 2A) or peak heart rate (179 ± 18 vs. 181 ± 11 vs. 179 ± 15 bpm, P = 0.582) for 8 v 8, 5 v 5 and 3 v 3, respectively. The mean heart rates corresponded to 86 ± 9, 89 ± 6 and 87 ± 5% HRpeak, respectively. No significant difference was found in blood lactate for 8v8, 5v5 and 3v3 (3.4 ± 2.3 vs. 2.4 ± 1.5 vs. 2.8 ± 1.9 mmol · L^-1^ respectively, P = 0.201 , Fig. 2B).

### Positional differences

No significant differences were found between D and M/F in any heart rate measures, blood lactate concentration or any of the variables describing movement patterns except 26–29% higher distance covered with 7–14 km · h^-1^ in M/F than D in 5 v 5 and 8 v 8 (P < 0.05) but not 3 v 3 (11%, P = 0.295).

### Correlations between game formats

**FIG. 3:**
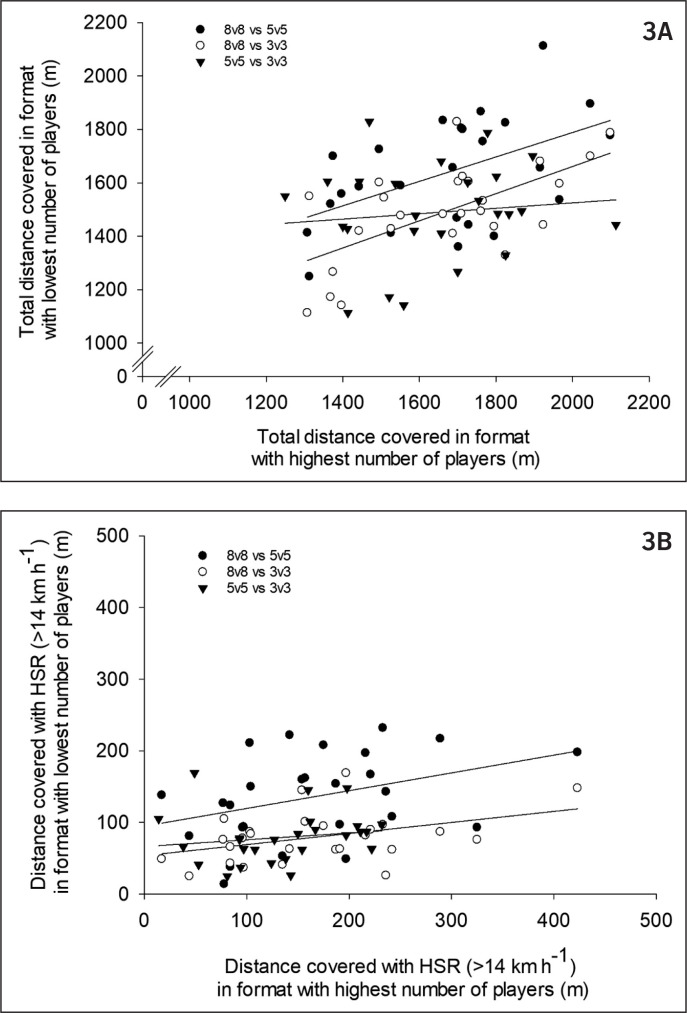
Correlations between A) total distance covered, and B) distance covered with high-speed running (HSR) with speed > 14 km · h^-1^ during 3 v 3, 5 v 5 and 8 v 8.

No significant correlations were found between 3 v 3 and 5 v 5 in any of the variables describing movement pattern. Total distance covered during 8 v 8 correlated moderately with 5 v 5 (r = 0.51, P < 0.05) and strongly with 3 v 3 (r = 0.63, P < 0.001 Fig. 3A). Similar moderate to large correlations between 8 v 8 and 5 v 5 and 3 v 3 (r = 0.37–0.67, P < 0.05) were found for distance within speed zones below 21 km · h^-1^ as well as HSR (r = 0.38 and r = 0.40, P < 0.05, respectively , Fig. 3B). Distance covered with VHSR or sprinting was not correlated between the three game formats. No correlations were found for peak speed, but number of intense accelerations was moderately correlated between 8 v 8 and 5 v 5 (r = 0.44, P < 0.05) and 8 v 8 and 3 v 3 (r = 0.46, P < 0.05).

Large to very large correlations were found for mean HR and peak HR between the three game formats (r = 0.57–0.84, P < 0.001), whereas no correlations were found for blood lactate (P > 0.45).

## DISCUSSION

The major finding of this study was that external load increased with concomitantly increasing number of players and absolute pitch size keeping area per player consistent, with no difference between game formats in internal load. Heart rate was strongly correlated between game formats, whereas no correlations were found for blood lactate. External load variables were not correlated between 5 v 5 and 3 v 3, whereas distance covered in total and in several speed zones below 21 km · h^-1^ as well as intense accelerations were correlated between 8 v 8 and the two other game formats. No position-specific differences were observed across all three game formats except greater distance covered for midfielders and forwards than defenders in 7–14 km · h^-1^ in 8 v 8 and 5 v 5 but not 3 v 3.

A 8–10% greater total distance covered during 8 v 8 and 5 v 5 than 3 v 3 was found (Fig. 1A), and moderate effects of game format were found on distance covered with HSR (Fig. 1B) and VHSR as greater distances in the highest speed zones were observed in formats on the largest pitches with the highest number of players. [6, 11] A number of studies have found greater distance covered in the highest speed zones during larger sided games [13, 17, 25, 26], whereas others have found no effect [15, 27] or greater distances covered during games with fewer players [16, 28]. It seems logical that it is more difficult to reach high speeds and thereby accumulate distance in the highest speed zones on smaller pitches, and in line with the majority of the literature higher peak speeds were in the present study observed during game formats with larger pitches. Pitch size and number of players are, however, closely connected, as changing only one of the parameters causes a change in the relative pitch size (area per player). In the two studies by Randers and colleagues, movement pattern was investigated in 3 v 3, 5 v 5 and 7 v 7 SSG, adapting pitch size to the number of players [15] or playing on a fixed pitch size (40 × 20 m), thus increasing relative pitch size in game formats with fewer players [16]. In these studies, it was concluded that increasing area per player stimulates high-speed running and increasing total distance, whereas number of players and pitch size had no effect on distances covered if relative pitch size was kept constant. In the present study, pitch size was adapted to the number of players in order to keep relative pitch size constant, but greater distances were covered during games with the largest number of players and thus the largest pitches, in agreement with the majority of the literature. These differences between the studies may be due to differences in the bout duration and pacing strategies [29] or the level of players, as it has been shown that elite and amateur football players respond differently in several variables of movement pattern across various game formats [14].

Another important descriptor of external load in football is accelerations and decelerations, which has attracted increasing focus in recent years [30, 31]. When playing SSG with a small relative pitch size, players must make several intense accelerations and changes of direction in order to create distance to the opponent and thereby increase the possibility to receive the ball. In the present study, no differences between game formats were found in the number of intense accelerations or intense decelerations, which is similar to the study by Randers and colleagues [16]. However, most studies have found higher number of accelerations and decelerations in game formats with fewer players on smaller pitches [15, 25, 26, 28]. When playing with fewer players, the involvement in the play and the number of ball contacts increase [32], but if the relative pitch size increases when decreasing the number of players, as in the study by Randers and colleagues [16], free space and distance to opponents also increase, which may increase continuous running and decrease acceleration/deceleration type movements. In the present study, however, relative pitch size was kept constant; thus the differences may partly be due to differences in thresholds and categorization of intense accelerations [25]. Accelerations, decelerations, and changes of directions contribute substantially to the internal load [33]. E.g. similar mean and peak heart rate and blood lactate response to SSG on very small pitches (20 × 12 m) surrounded by boards compared to without boards have been observed even though distances covered with high-speed running (> 13 km/h) were up to 242% longer without than with boards [33]. It was suggested that the higher number of accelerations, decelerations and changes in direction found with than without boards compensated for the lower high-speed running distance, leading to a comparable heart rate response and blood lactate levels [33].

In the present study similar mean and peak HR were observed between game formats (Fig. 2A), which is similar to some studies [15, 26, 27, 34], whereas others have found increasing heart rates with decreasing pitch sizes and number of players [16, 19, 35]. Distances covered in total and with high speed were greater during games on larger pitches; thus it may seem surprising that no differences were found in heart rate response. As mentioned above, a number of studies have found that movement pattern switches from high-speed runs to a more acceleration/deceleration-based pattern, when pitch size becomes small. In the present study, no differences in intense accelerations and decelerations were observed between game formats; thus the similar internal load cannot be explained by increased numbers of accelerations and decelerations in game formats on the smallest pitches.

Mean and peak HR were 155–160 b.p.m. and 179–181 b.p.m., respectively [15, 16], corresponding to 86–89% of HRpeak, which is similar to what has been observed in other studies [6, 9, 11] [6, 9, 11]. It should be noted, however, that the relative mean heart rate values in the present study may have been overestimated as the peak HR was only ~180 b.p.m. An underestimation of peak HR has, however, no practical implication for comparing the three game formats, but the internal load is likely lower than observed in previous studies.

A possible lower internal load than observed in previous studies is also indicated by low blood lactate values (2.4–3.4 mmol · L^-1^ , Fig. 2B) compared to other studies showing blood lactate values of 4.5–7.4 mmol · L^-1^ [15, 16, 19, 26]. However, this difference may also be due to the level of players, as elite players were recruited in the present study whereas lower level players were recruited in the previous studies [15, 16, 19, 26]. A higher blood lactate response to various SSG may be expected in amateur players compared to professionals due to the difference in fitness level [14].

To improve physical performance, various formats of SSG are used during micro and macro cycles and the overall difference in response in various parameters is in general considered to apply for all players even though practitioners must be aware of several factors challenging the extensive use of SSG [8]. In the present study no correlation between 3 v 3 and 5 v 5 was found in any of the movement variables or blood lactate response but only heart rate variables, whereas 8 v 8 correlated with 5 v 5 and 3 v 3 in movement variables in the lowest speed categories as well as accelerations and heart rate variables. Hence, it is important to monitor individuals consistently, as both the external and internal loads vary markedly on an individual level between game formats especially in the variables related to the highest intensity actions [8]. Studies have also shown that drill-to-drill and match-to-match variation is high in the highest speed zones [36, 37]. Consequently, coaches should be aware of the different responses on an individual level when applying different game formats to manipulate individual physiological responses. In line with this, no positional differences were found for all values except running distance with 14–21 km/h in 8 v 8 and 5 v 5, although large positional differences are found in game demands [38–40].

## CONCLUSIONS

Greater distance was covered in total and with high-speed running (> 14 km · h^-1^) and very high-speed (21 km · h^-1^), and peak running speed was higher in SSG formats with more players and on larger pitch sizes for Chinese elite youth football players. However, the higher external load did not lead to differences in internal load measured by heart rate and blood lactate. Some correlations were found between 8 v 8 and the two other game formats in internal and external load variables, but not between 3 v 3 and 5 v 5.
